# Mitochondrial Ion Channels of the Inner Membrane and Their Regulation in Cell Death Signaling

**DOI:** 10.3389/fcell.2020.620081

**Published:** 2021-01-05

**Authors:** Andrea Urbani, Elena Prosdocimi, Andrea Carrer, Vanessa Checchetto, Ildikò Szabò

**Affiliations:** ^1^Department of Biomedical Sciences, University of Padova, Padua, Italy; ^2^Department of Biology, University of Padova, Padua, Italy

**Keywords:** mitochondria, ion channel, cell death, cell signaling, apoptosis

## Abstract

Mitochondria are bioenergetic organelles with a plethora of fundamental functions ranging from metabolism and ATP production to modulation of signaling events leading to cell survival or cell death. Ion channels located in the outer and inner mitochondrial membranes critically control mitochondrial function and, as a consequence, also cell fate. Opening or closure of mitochondrial ion channels allow the fine-tuning of mitochondrial membrane potential, ROS production, and function of the respiratory chain complexes. In this review, we critically discuss the intracellular regulatory factors that affect channel activity in the inner membrane of mitochondria and, indirectly, contribute to cell death. These factors include various ligands, kinases, second messengers, and lipids. Comprehension of mitochondrial ion channels regulation in cell death pathways might reveal new therapeutic targets in mitochondria-linked pathologies like cancer, ischemia, reperfusion injury, and neurological disorders.

## Introduction

Mitochondria are dynamic organelles that are primarily recognized as the “powerhouse” of the cell, where the energy stored in nutrients is converted to ATP molecules through the oxidative phosphorylation (OXPHOS). Besides the production of ATP, mitochondria also play fundamental roles in other cellular functions, including metabolism of fatty/amino acids (Spinelli and Haigis, 2018), Ca^2+^ homeostasis (De Stefani et al., 2016), thermogenesis (Chouchani et al., 2019), redox signaling (Zorov et al., 2014), and cell death (Galluzzi et al., 2016).

Regulated cell death is critical to development, tissue homeostasis, and removal of cells with abnormal behavior. Some key cell death controlling proteins include caspases, Bcl-2 family proteins, death receptors, RIP kinases, inhibitor of apoptosis proteins (IAPs), Endonuclease G, Apoptosis Inducing Factor (AIF), Caspase-Activated DNAase, Apaf-1, SMAC/DIABLO, and HtrA2/OMI. In several studies, various ion channels emerge as crucial regulators of various forms of cell death [for reviews see e.g., (Bortner and Cidlowski, 2014; Kondratskyi et al., 2015; Leanza et al., 2015; Fricker et al., 2018; Bachmann et al., 2019)].

**FIGURE 1:**
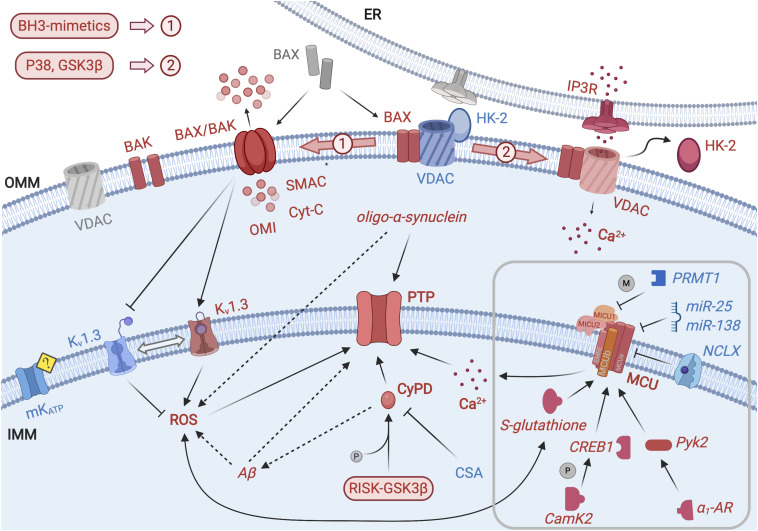
Permeability transition, MCU modulation and mitochondrial regulation of apoptosis. Scheme representing the main ion channels and molecular actors responsible for mitochondrial regulation of apoptosis. Promoters of PT and cell death are depicted as red elements, while blue elements represent inhibitors of PT/cell death or pro-survival factors; molecular actors in a neutral/inactive state are represented as gray elements. PTP is located at the IMM and is the center of converging signaling pathways regulating PT. PTP opening is triggered by Ca^2+^ and facilitated by mitochondrial ROS and by the binding of CyPD. The latter is favored by CyPD phosphorylation by GSK3β and by RISK kinases; conversely CsA-driven detachment of CyPD has an inhibitory effect on PTP. IMM-localized MCU allows Ca^2+^ internalization in the mitochondrial matrix and, in turn, PTP opening. Kv1.3 indirectly regulates ROS formation, since mitochondrial accumulation of the latter is promoted when the former is inhibited. Channel activity of ATP-dependent K^+^ channel (mK_*ATP*_) has recently been shown to exert a pro-survival and anti-apoptotic action, although through unclear mechanisms. Aβ and oligomers of α-synuclein probably exert both a direct and ROS-mediated action on the PTP, promoting its opening, but the molecular mechanism is still lacking. The main actors of MOMP, located in the OMM, are in an active equilibrium between inactive and pro-apoptotic state. In particular, in the presence of pro-apoptotic stimuli, BAX localizes in the OMM and forms BAX/BAK complexes, allowing the release in the cytosol of pro-apoptotic factors, initiating the caspase cascade. BAX/BAK activation is promoted by BH3-mimetics. Moreover, the interaction between OMM-localized BAX and Kv1.3 in the IMM causes the inhibition of the latter, with a PT-promoting effect. VDAC and HK-2 are associated at the interface with the ER but the activation of p38 and GSK3β stimulates the detachment of HK-2, leading to cell death via Ca2+ release through IP3R. The MCU located in the IMM is responsible for Ca^2+^ uptake. In the gray square, modulation of the MCU activity is depicted in detail. The expression of the MCU subunits is post-transcriptionally down-regulated by miR-25 and miR-138. ROS regulate MCU activity; especially, Cys-97 in the MCU sequence has been identified as a target of mROS and undergoes S-glutathionylation. Moreover, MCU is positively regulated by CamK2: activated CaMK2 promotes CREB phosphorylation and enhances its gene transcriptional function. Furthermore, MCU opening is modulated by Pyk2: α_1_-AR signaling leads to translocation of activated Pyk2 from the cytosol to the mitochondrial matrix; this event accelerates mitochondrial Ca^2+^ uptake increasing mitochondrial ROS production and promoting PTP opening and apoptotic signaling. MCU is controlled by PRMT1 that asymmetrically methylates MICU1 subunit, resulting in decreased Ca^2+^ sensitivity. Finally, NCLX activates mitochondrial Ca^2+^ extrusion via a protein kinase A-mediated phosphorylation-dependent manner.

The role of mitochondria in cell death is linked to apoptosis, where mitochondrial outer membrane permeabilization (MOMP) originates a signaling cascade leading to cell death (Bock and Tait, 2020). MOMP is initiated by the formation of macropores in the outer mitochondrial membrane (OMM) (Szabo and Zoratti, 2014), allowing the release of soluble proteins from the mitochondrial intermembrane space (IMS) in a process regulated by the B cell lymphoma 2 (BCL-2) protein family. The proteins comprised in this family can be subdivided into three types: anti-apoptotic proteins (BCL-2, BCL-W, BCL-X_*L*_, A1, and MCL1), pro-apoptotic proteins (BAK, BAX, and BOK), and pro-apoptotic BH3-only proteins (BID, BIM, BAD, BIK, BMF, HRK, NOXA, and PUMA) (Bock and Tait, 2020). In the presence of pro-apoptotic stimuli, activation of BH3-only proteins leads to activation of BAK, BAX, and BOK (Correia et al., 2015). These active pro-apoptotic proteins undergo hetero-homo oligomerization forming dynamic macropores in the OMM through which IMS proteins are released into the cytosol (McArthur et al., 2018) (Figure 1). Among IMS proteins, cytochrome *c* (an essential component of the electron transport chain), when released into the cytosol, contributes to the formation of apoptosomes, which initiate the caspase cascade. Also, MOMP causes the release of other pro-apoptotic proteins, including SMAC/DIABLO and HtrA2/OMI [for a recent review see Bock and Tait (2020)].

In addition to the aforementioned factors, in several studies, ion channels, including potassium, calcium, sodium, and chloride channels, have been pointed out as crucial regulators of apoptotic cell death (Razik and Cidlowski, 2002; Lang et al., 2005; Leanza et al., 2013). Ion channels are integral membrane proteins mediating ionic fluxes, driven by electrochemical gradient, through biological membranes. Ion channels allow ion compartmentalization and regulation of membrane potential and cell volume. Their opening and closing are often regulated by many factors, including chemical signals and mechanical stimulation (O’Rourke, 2007). In mitochondria, ion channels, present both in the OMM and in the inner mitochondrial membrane (IMM), are actively involved in the regulation of several mitochondrial processes such as regulation of membrane potential and ROS release as well as, volume regulation (O’Rourke, 2007; Szabo and Zoratti, 2014).

Many recent reviews describe the role of mitochondrial ion channels in cell death, especially in apoptosis and necrosis (Biasutto et al., 2016; Krabbendam et al., 2018; Magri et al., 2018; Szewczyk et al., 2018; Peruzzo and Szabo, 2019). In this minireview, we provide an updated overview specifically on the link between the complex regulation of the IMM channels and death induction, with special attention given to the permeability transition pore (PTP) and the mitochondrial calcium uniporter, two master regulators of cell fate.

## The Permeability Transition Pore

### Permeability Transition: A Mitochondrial Catastrophe Regulated by a Mitochondrial Megachannel Whose Molecular Identity Is Still Debated

Even when signaling pathways responsible for MOMP initiation are not fully activated, cytochrome *c* and other IMS proteins can be released in the cytosol via the rupture of the OMM due to mitochondrial swelling initiated by a process called “permeability transition” (PT) (Petronilli et al., 2001). PT is defined as a sudden increase in IMM permeability to ions and other solutes up to 1.5 kDa, leading to mitochondrial depolarization, cessation of ATP synthesis, and eventually to cell death. This process occurs during profound stress that are often linked to pathological conditions. PT is a regulated and reversible process, requiring matrix Ca^2+^ accumulation and is caused by the opening of a channel called PTP. PTP opening is a highly regulated event, being facilitated by binding of cyclophilin D (CyPD), free fatty acids, Pi (in mammalian mitochondria), accumulation of matrix calcium, of reactive oxygen species (ROS), or by low transmembrane potential. PTP is inhibited by adenine nucleotides and Mg^2+^, detachment of CyPD by cyclosporin A (CsA), and mildly acidic matrix pH [for review see e.g., (Bernardi et al., 2015)].

The idea that PT could be due to a regulated pore (Hunter and Haworth, 1979) was corroborated by the discovery of a Ca^2+^-activated, unselective, high-conductance channel in the IMM with the same characteristics of PTP (Szabo and Zoratti, 1992). This channel, named mitochondrial megachannel (MMC) or multi-conductance channel (MCC), has been deeply characterized using patch clamp on mitoplasts: typically, MMC opens at potential (*V*) values near to 0 and displays a very high maximal conductance (1.3–1.5 nS) with a large number of lower conductance substates (Kinnally et al., 1989; Petronilli et al., 1989). Ca^2+^ drives MMC activation in the sub-mM range on the matrix side while inhibition takes place at mildly acidic matrix pH, and in the presence of divalent cations other than Ca^2+^ as well as of CSA.

**FIGURE 2:**
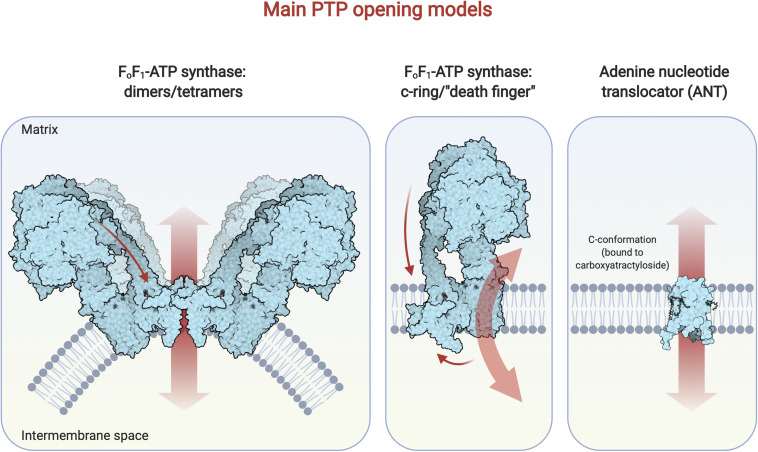
Main PTP opening models. Here the most recent and supported models for PTP opening are depicted. On the left: the F_o_F_1_-ATP synthase dimer/tetramer hypothesis postulates that the pore forms within the membrane-embedded domain, comprising the e and g subunits at the dimer/tetramer interface; a conformational change is induced by Ca^2+^ binding to the F_1_ portion of the enzyme and is transmitted (arrow) via the peripheral stalk (Giorgio et al., 2018). The diagram was drawn on the basis of a recent crystal structure (Spikes et al., 2020) of dimeric bovine F-ATP synthase. In the middle: the c-ring hypothesis proposes that the pore opens within the c-ring after the Ca^2+^- dependent dissociation of the F_1_ domain from F_o_ (Alavian et al., 2014). The “death finger” hypothesis (Gerle, 2016, 2020) posits that Ca^2+^ binding in the F_1_ domain determines a conformational change in the region that is transmitted along the peripheral stalk (upper arrow), leading to the displacement of subunits e and 6.8 PL in the F_o_ portion; this, in turn, allows the removal of the lipid plug from the c-ring leading to PTP opening. The diagram was drawn on the basis of the same crystal structure referred above (Spikes et al., 2020). On the right: the ANT hypothesis [extensively reviewed in Bround et al. (2020)] proposes that a Ca^2+^-dependent conformational change of the ANT leads to PTP opening. The ANT c-state diagram was adapted from a crystal structure (Nury et al., 2005) of ANT in association with its selective inhibitor, carboxyatractyloside, that has been recognized as a PTP-inducer.

The molecular identity of PTP has long been elusive; here, we briefly mention the potential consensus candidates for pore formation: the adenine nucleotide translocator (ANT) and F_o_F_1_ -ATP synthase. ANT is a family of IMM transmembrane proteins whose physiological role is to exchange matrix ATP for cytosolic ADP. ANT was the first molecular candidate to be proposed for PTP formation (Figure 2) on the basis of pharmacological (Hunter and Haworth, 1979) and electrophysiological (Brustovetsky and Klingenberg, 1996) considerations. On the other hand, mitochondria from ANT1/2-null mice underwent Ca^2+^-dependent PTP opening, although with higher Ca^2+^ level required for PT initiation, compared to wild-type (WT) (Kokoszka et al., 2004). Likewise, mitochondria from *Ant1*^–/–^, *Ant2*^–/–^, and *Ant4*^–/–^ triple KO mice required a higher matrix Ca^2+^ load for PTP opening, that still occurred and was inhibited by both CsA and by ablation of CyPD (Karch et al., 2019). These findings indicate that PT can take place in the absence of any ANT isoforms, although they suggest that ANT can team up with the pore to ensure higher order regulation.

The other, best-accredited candidate is the mitochondrial F_o_F_1_-ATP synthase (or F-ATP synthase), a key actor of OXPHOS that converts transmembrane proton motive force into chemical energy via mechanical rotation, leading to the synthesis of ATP molecules (Carraro et al., 2020). The F-ATP synthase hypothesis of pore formation may appear quite unlikely, given the lack of obvious structural evidence of an ion permeation pathway other than H^+^ and the chemiosmotic principle for which a tightly coupled F-ATP synthase is essential to power ATP generation. Despite this, evidence, mainly based on a combination of pharmacology, molecular biology, and electrophysiology were obtained, pointing to a crucial role of this enzyme in PTP formation (Giorgio et al., 2013; Antoniel et al., 2018; Carraro et al., 2018). However, Walker’s group provided opposing evidence: PTP formation was assessed in HAP1 haploid human cells devoid of individual subunits of the peripheral stalk of F-ATP synthase and, as a consequence, lacking a fully functional enzyme; those cells underwent Ca^2+^-dependent swelling, although with a slower kinetics compared to WT, and showed CsA-sensitive Ca^2+^-induced Ca^2+^ release (He et al., 2017; Carroll et al., 2019). Overall, these results were interpreted contrasting with the idea that PTP originates from F-ATP synthase (Walker et al., 2020) and alternative hypotheses have been raised (Carraro and Bernardi, 2020; Carraro et al., 2020). Importantly, recent data showed that highly purified F-ATP synthase can give rise to MMC-like activity upon reconstitution into artificial membranes (Mnatsakanyan et al., 2019; Urbani et al., 2019). In support of the crucial role of subunit e for PTP opening, emerging from recent studies (Carraro et al., 2018), and of the death finger model (Gerle, 2016, 2020), new Cryo-EM data of the enzyme exposed to calcium revealed that retraction of subunit e led to a gradually disassembled c-ring, suggesting pore formation by spaced out c subunits (Pinke et al., 2020; Figure 2).

### PTP in Physiopathology

Prolonged PTP opening has long been known to be relevant in a high number of pathological conditions - heart injury was the first to be recognized, based on the protective effect of CsA against ischemia-reperfusion injury (Duchen et al., 1993; Griffiths and Halestrap, 1993). Apart from heart injury, several neurodegenerative diseases share calcium mishandling and increased ROS production in neurons, linked to persistent PTP opening (Mattson et al., 2008). Those diseases include excitotoxicity (Pivovarova and Andrews, 2010), Huntington’s disease (Quintanilla et al., 2017) [however, several works from Brustovetsky’s group indicates that Ca^2+^ mishandling and mitochondrial function are not impaired in this pathology (Pellman et al., 2015; Hamilton et al., 2016, 2017, 2019)], Alzheimer’s disease (Du et al., 2011), and Parkinson’s disease (Martin et al., 2014). Furthermore, strong evidence supports a role for CyPD and PTP in the pathology of the Bethlem or Ullrich type muscular dystrophies, consequences of deficiency of collagen VI, delta-sarcoglycan, or laminin-2 (Millay et al., 2008; Palma et al., 2009). Recent studies point to PTP implication in bone loss associated with osteoporosis (Shum et al., 2016) and suggest that pore inhibition could improve bone fracture repair (Shares et al., 2020).

Despite the defined role of prolonged pore openings in pathologies, transient PTP openings have early been hypothesized not to be detrimental (Gunter and Pfeiffer, 1990; Zoratti and Szabo, 1995), and later they were shown to be relevant in the physiological Ca^2+^ homeostasis in several experimental settings (Hüser and Blatter, 1999; Petronilli et al., 1999; Hausenloy et al., 2004; Agarwal et al., 2017; Ying et al., 2018).

### PTP Opening Modulation by Intracellular Factors in the Context of Cell Death

Given the fundamental role of PTP in cell death and, consequently, in various pathologies, understanding its regulation under physiopathological conditions is of utmost importance. PTP can be regulated by ions, small molecule drugs [for a recent review see e.g., (Leanza et al., 2018)], lipids (Brini et al., 2017) as well as proteins (see below). Besides requiring Ca^2+^, PTP opening is induced by ROS, by-products of OXPHOS. Indeed, accumulation of mitochondrial ROS during reperfusion after ischemia in cardiomyocytes induces PTP openings that can be suppressed by antioxidants administration (Assaly et al., 2012). Modulation of PTP by ROS seems to play a role also in Parkinson’s disease: redox-active α-synuclein oligomers, able to impair mitochondrial function and cause PTP opening, have been shown to trigger ROS generation and to directly oxidize a PTP candidate, F-ATP synthase (Ludtmann et al., 2018).

Although its physiological role remains elusive, CyPD, a highly conserved mitochondrial peptidyl-prolyl cis-trans isomerase, has long been known to be a sensitizer of the PTP to Ca^2+^ and ROS and the mediator of the action of the typical PTP inhibitor, CsA (Giorgio et al., 2010). Several CyPD post-translational modifications (PTMs) correlating with pore regulation have been identified by recent studies (Amanakis and Murphy, 2020). One of the most relevant PTMs pathway is the RISK-GSK3β-CyPD axis: a set of kinases known as RISK (reperfusion injury salvage kinases, including AKT, cdk5, ERK, PKA, PKC, and PKG, – constitutively active Ser/Thr protein kinase that phosphorylates, among other substrates, CyPD, favoring its interaction with PTP (Rasola et al., 2010; Figure 1). p38, as well as GSK3β, contribute to the regulation of PTP also by another mechanism: they control the mitochondrial localization of Isoform 2 of the glycolytic enzyme hexokinase (HK2) in a still unclarified way (Bikkavilli et al., 2008). Activation of p38 or GSK3β initiates mitochondrial dissociation of HK2, which, in turn, promotes cell death (Tanaka et al., 2018; Ciscato et al., 2020), presumably due to a burst of Ca^2+^ released from the endoplasmic reticulum (ER) via IP3R, leading to a massive Ca^2+^ uptake into the mitochondrial matrix that in turn opens the PTP (Ciscato et al., 2020). In cancer cells, elevated levels of OMM-bound HK2 results in evasion of PTP-dependent apoptosis (Marchi et al., 2019).

CyPD can also interact with amyloid-β protein (Aβ), resulting in enhanced ROS levels that ultimately triggers PTP opening (Du et al., 2008). Indeed, CyPD deficiency alleviates mitochondrial stress and neuronal damage (Du et al., 2011). Aβ *per se* may act directly on PTP, since it can interact with the oligomycin sensitivity conferring protein (OSCP) subunit of F_o_F_1_-ATP synthase, inducing the formation of an Aβ-OSCP complex; OSCP sequestration, in turn, disrupts F_o_F_1_-ATP synthase, leading to dampened ATP production, increased oxidative stress, and PTP activation (Beck et al., 2016).

Interestingly, PTP regulation by CyPD/CsA has also been linked to viral infection. Apoptosis represents an important defense mechanism for host cells in the case of many viruses (SADS-COV, hepatitis B/C virus, PDCoV, SARS-COV, and MERS) (Zhang et al., 2020). Virus infections promote BAX translocation and, in turn, MOMP and PTP activation, eventually leading to cytochrome *c* release (Lee and Lee, 2018); CsA treatment efficiently inhibited both virus replication/infection and host cell death. Despite this, it is still unclear if CsA treatment prevents apoptosis by sequestering CyPD (with direct implication in PTP opening) or CyPA (affecting virus replication and BAX translocation) (Glowacka et al., 2020; Molyvdas and Matalon, 2020).

Permeability transition pore opening is facilitated in the presence of variants of Apolipoprotein L1 (APOL1) associated with kidney disease and linked to mitochondrial dysfunction. Whereas APOL1 is mostly monomeric, APOL1 risk-variants display self-aggregation in higher-order oligomers that can physically interact with putative PTP components (F-ATP synthase and ANT2), triggering PTP opening and cell death (Shah et al., 2019).

Finally, it has to be mentioned that the F-ATP synthase can be physiologically regulated also by the ATPase Inhibitory Factor 1 that mediates cell survival by promoting mild ROS release resulting from metabolic shift due to inhibition of F-ATP synthase (Martínez-Reyes and Cuezva, 2014).

## The Mitochondrial Calcium Fluxes and Their Regulation in Cell Death

### Mitochondrial Calcium Uniporter Complex

Mitochondrial Ca^2+^ channels participate in many intracellular signaling pathways both in physiological and in pathological conditions and crucially balance cell life versus death (Giacomello et al., 2007; Duchen et al., 2008). Indeed, as mentioned above, calcium overload in the mitochondria matrix leads to persistent PTP opening and eventually to cell death. Therefore, matrix calcium level has to be highly regulated by modulating the activity of IMM calcium-permeable ion channels and transporters (Rizzuto et al., 2012; Zavodnik, 2016).

Mitochondria can rapidly achieve a high [Ca^2+^]_*matrix*_ thanks to the presence of a huge driving force generated by a ΔΨm of −180 mV under physiological conditions, and to the tight contact between the ER and mitochondria that allows direct channeling for Ca^2+^ (Rizzuto et al., 1998; Naon and Scorrano, 2014; Giorgi et al., 2015) (see Figure 1).

Calcium entry is primarily mediated by the mitochondrial calcium uniport (MCU) complex (Baughman et al., 2011; De Stefani et al., 2011), which is able to sense the Ca^2+^ signals originating from the ER, while release takes place through the Na^+^/Ca^2+^ exchanger NCLX (Palty et al., 2010). At the current stage, the mammalian MCUC appears to consist of at least of the pore-forming protein MCU, a dominant-negative MCU paralog (MCUb), the essential MCU regulator (EMRE), the regulatory MICU proteins (MICU1-3), and possibly, the mitochondrial calcium uniport regulator 1 (MCUR1) (for reviews see e.g., (Marchi and Pinton, 2014; De Stefani et al., 2015; Wagner et al., 2016; Cui et al., 2019). Interestingly, MCU also conducts Mn^2+^, depending on the presence of MICU1 (Kamer et al., 2018).

### Regulation of MCU and of NCLX Affecting Cell Death

Mitochondrial calcium uniport has a documented, crucial role in both proliferation and apoptosis [for reviews see e.g., (De Stefani et al., 2015; Bustos et al., 2017; Cui et al., 2017; Bachmann et al., 2019)]. The expression of the MCU subunit can be post-transcriptionally down-regulated by several small non-coding regulatory RNAs (miR), miR-25, and miR-138 (Marchi et al., 2013; Hong et al., 2017; Jaquenod De Giusti et al., 2018; Figure 1). The miRs drastically decrease MCU protein levels, blocking thus mitochondrial Ca^2+^ uptake without affecting [Ca^2+^]_*C*_ and [Ca^2+^]_*ER*_, causing reduced apoptosis in cancer cells. These miRs also affect the expression of proapoptotic proteins, like Bim (Zhang et al., 2012), TRAIL (Razumilava et al., 2012), and PTEN (Poliseno et al., 2010).

Similarly to PTP, MCU activity can be regulated by ROS: a highly conserved Cys-97 at the matrix side of MCU was observed to be S-glutathionylated under oxidative stress, leading to enhanced MCU tetramerization and channel activity that, in turn, exacerbates mitochondrial Ca^2+^ overload and triggers cell death (Dong et al., 2017).

Other types of PTMs can also regulate MCU. One of the first reported cases envisioned Ca^2+^/calmodulin-dependent protein kinase 2 (CaMK2) as a regulator of MCU, however, this finding has been challenged (Joiner et al., 2012, 2014; Fieni et al., 2014). Moreover, MCU is regulated by the proline-rich tyrosine kinase 2 (Pyk2), accelerating mitochondrial Ca^2+^uptake *via* Pyk2-dependent MCU phosphorylation and tetrametric MCU channel pore formation under α_1_-adrenoceptor (α_1_-AR) signaling.

Furthermore, mitochondrial Ca^2+^ uptake is controlled by the modification of MCU regulators. For example, the protein arginine methyltransferase 1 (PRMT1) methylates MICU1 subunit, decreasing its Ca^2+^ sensitivity, thus resulting in reduced calcium uptake into the matrix (Madreiter-Sokolowski et al., 2016). Interestingly, it has been observed that the uncoupling proteins 2/3, previously shown to affect mitochondrial calcium handling, were able to ensure the sensitivity of MICU1 to calcium even upon increased methylation activity (Madreiter-Sokolowski et al., 2016). However, this finding does not explain how upregulation of UCP2 expression can protect mitochondria against calcium overload and cells from apoptosis (Pan et al., 2018). Independently of this, a similar interplay between ANT and F-ATP synthase may exist since the mitochondrial lysine (K)-specific methyltransferase (KMT) FAM173B targets the c-subunit of mitochondrial ATP synthase while FAM173A methylates ANT2 and 3 (Małecki et al., 2019).

As to NCLX, a recent study illustrates the physiological relevance of its post-translational regulation: adrenergic stimulation of brown adipose tissue was shown to activate mitochondrial Ca^2+^ extrusion via the mitochondrial NCLX in a protein kinase A-mediated phosphorylation-dependent manner, in order to prevent cell death despite the sharp increase of [Ca^2+^]_*m*__*atrix*_ during thermogenesis (Assali et al., 2020). Inhibition of NCLX by the microtubule-associated tau protein implicated in the tauopathies was instead linked to increased death in neurons (Britti et al., 2020). Recent pieces of evidence from NCLX KO mice suggest an anti-apoptotic role exerted by NCLX by preventing mitochondrial Ca^2+^ overload (Luongo et al., 2017; Assali et al., 2020).

## Modulation of Other IMM Ion Channels Affects Cell Survival

Many other ion channels in the IMM have been linked to cell survival/cell death, including the mitochondrial counterparts of potassium channels TASK-3 (Nagy et al., 2014), calcium-activated potassium channels (Dolga et al., 2013; Krabbendam et al., 2018), the recently identified ATP-dependent K^+^ channel (Laskowski et al., 2019; Paggio et al., 2019) and voltage-gated K^+^ channels (Szabo et al., 2005; Testai et al., 2015) and the uncoupling proteins (Adams et al., 2010; Pitt, 2015). Unfortunately, only limited information is available about the intracellular regulation of these channels, apart the classical ligands known to modulate these channels such as ATP, protons, fatty acids and calcium. One exception is the voltage-gated Kv1.3, shown to interact with OMM-inserted BAX (Szabo et al., 2008) via critical lysine residues during apoptosis (Szabo et al., 2011; Figure 1). The channel becomes inhibited, and via interaction with complex I, Kv1.3 inhibitors trigger ROS release (Peruzzo et al., 2020), leading to PTP opening. This observation inspired the design of a mitochondriotropic inhibitor of the channel that triggered apoptosis and drastically reduced tumor volume of both melanoma and pancreatic ductal adenocarcinoma *in vivo*. Hopefully future research will identify ways to target other channels that are differentially expressed in various tissues and cancer types.

## Conclusion and Future Outlook

In conclusion, advances in the genetic identification of IMM channel components, along with the availability of Mitocarta (Pagliarini et al., 2008) and novel tools to identify interaction partners in intact cells (Branon et al., 2018), will certainly revolutionize the field of mitochondrial ion channels. As illustrated above, considerable new information allowed to link pathways/proteins that regulate IMM channels to cell death/survival signaling during the last decade. Since factors that regulate mitochondrial calcium accumulation play a crucial role in this context, understanding of the relation between channel modulation and localized [Ca^2+^] changes, thanks to novel, sub-mitochondrial targeted Ca^2+^ sensors (Waldeck-Weiermair et al., 2019), may further widen our view. Similarly, measurement of simultaneous, real-time dynamics of ATP and ROS in mitochondria *in vivo* (van Hameren et al., 2019) may provide important insights to integrate IMM (and OMM) channels into death signaling pathways. As a challenging future outlook, we may envision constructing artificial, light-gated ion channels (Cosentino et al., 2015) targeted to mitochondria.

## Author Contributions

All authors contributed to the writing of the manuscript.

## Conflict of Interest

The authors declare that the research was conducted in the absence of any commercial or financial relationships that could be construed as a potential conflict of interest.
